# Lycopene supplementation prevents reactive oxygen species mediated apoptosis in Sertoli cells of adult albino rats exposed to polychlorinated biphenyls

**DOI:** 10.2478/intox-2013-0015

**Published:** 2013-06

**Authors:** Gunasekaran Krishnamoorthy, Kandaswamy Selvakumar, Prabhu Venkataraman, Perumal Elumalai, Jagadeesan Arunakaran

**Affiliations:** 1 Department of Biochemistry, Asan Memorial Dental College & Hospital, Asan Nagar, Chengalpet 603105, India; 2 Department of Endocrinology, Dr. ALM Post Graduate Institute of Basic Medical Sciences, University of Madras, Chennai 600113, India; 3 Department of Medical Research, SRM Medical College & Hospital, SRM University, Kattankulathur 603203, India

**Keywords:** apoptosis, oxidative stress, caspases, lycopene, polychlorinated biphenyls, Sertoli cells

## Abstract

Sertoli cell proliferation is attenuated before attaining puberty and the number is fixed in adult testes. Sertoli cells determine both testis size and daily sperm production by providing physical and metabolic support to spermatogenic cells. Polychlorinated biphenyls (PCBs) exposure disrupts functions of Sertoli cells causing infertility with decreased sperm count. On the other hand, lycopene is improving sperm count and motility by reducing oxidative stress in humans and animals. Hence we hypothesized that PCBs-induced infertility might be due to Sertoli cell apoptosis mediated by oxidative stress and lycopene might prevent PCBs-induced apoptosis by acting against oxidative stress. To test this hypothesis, animals were treated with vehicle control, lycopene, PCBs and PCBs + lycopene for 30 days. After the experimental period, the testes and cauda epididymidis were removed for isolation of Sertoli cells and sperm, respectively. We observed increased levels of oxidative stress markers (H_2_O_2_ and LPO) levels, increased expression of apoptotic molecules (caspase-8, Bad, Bid, Bax, cytochrome C and caspase-3), decreased anti-apoptotic (Bcl2) molecule and elevated apoptotic marker activity (caspase-3) in Sertoli cells of PCBs-exposed animals. These results were associated with decreased sperm count and motility in PCBs exposed animals. On the other hand, lycopene prevented the elevation of Sertoli cellular apoptotic parameters and prevented the reduction of sperm parameters (count and motility). The data confirmed that lycopene as an antioxidant scavenged reactive oxygen substances, prevented apoptosis, maintained normal function in Sertoli cells and helped to provide physical and metabolic support for sperm production, thereby treating infertility in men.

## Introduction

Polychlorinated biphenyls (PCBs) are used as an insulating material in electric equipment and more stable organic molecules were widely used during the 1940s. Exposure to PCBs remains an important public health issue and the identification of potential therapeutic approach to protect against the reproductive toxicity of these environmental contaminants is of significant interest. Dietary lycopene is a highly efficient antioxidant and has O_2_^**•**–^ and HO**•** scavenging capacity (Cohen 2002; Tapero *et al.*, 2004). Due to its persistence and lipophilicity, it accumulates in biologic tissue and bioconcentrates at successively higher levels of the food chain (DeCastro *et al.*, 2006).

PCBs-induced cytotoxicity has been implicated in ROS generation due to the depletion of antioxidants in Leydig cells (Murugesan *et al.*, 2007a) and Sertoli cells (SCs) (Senthilkumar *et al.*, 2005). PCBs induce cytochrome P450s as a possible source of ROS (Schlezinger *et al.*, 2000) or alternatively, PCB derivatives undergo redox cycling with the formation of ROS like O_2_^**•**–^, HO^**•**^ and H_2_O_2_, thus becoming another source of oxidative stress (McLean *et al.*, 2000). ROS are thought to contribute to LPO (Hochstein & Ernster, 1963), DNA damage (Kasai *et al.*, 1986) and protein degradation (Griffith *et al.*, 1988). On the other hand, ROS have also been demonstrated to perform certain functions in the early stages of apoptosis. Apoptosis can be induced through two distinct pathways; one involves the ligation of the TNF/Fas-receptor with its ligand, which then followed by caspase-8 activation. This in turn either directly activates caspase-3, or causes it to merge with the mitochondrial pathway via cleavage of the Bcl-2 family member, Bid. The other pathway is the mitochondria mediated caspase-9 activation pathway. Both pathways converge in caspase-3, culminating in cell death. ROS are generated in the mitochondria and from other sources and inflict serious damage to lipids, proteins and DNA (Orrenius, 1992).

SCs have long been known to be the targets for various toxicants (Steinberger & Klinefelter, 1993), including polychlorinated biphenyls (Syed *et al.*, 1997). Sertoli cells are recognized as secretory cells, which have been shown to be responsible for the biosynthesis and release of most proteins found in the lumen of seminiferous tubules. In the mammalian testis, SCs play a key role in the initiation and maintenance of spermatogenesis (Carreau *et al.*, 1994). Lactate is a preferential energetic substrate for germ cells because of the following three reasons: 1) the inability of germ cells to produce energy by utilizing glucose, 2) preference for lactate as an energy source, and 3) the ability of lactate production in huge amount by Sertoli cells for the metabolic co-operation between Sertoli cell and germ cell (Nehar *et al.*, 1998). γ-glutamyl transpeptidase (γ-GT) is a membrane-bound enzyme and is considered a functional marker of Sertoli cells. It catalyzes the transfer of the γ-glutamyl group from glutathione to peptides, amino acids and water.

Investigations from several research laboratories revealed that one individual Sertoli cell nurses a clone of developing germ cells (Dubois & Callard, 1990). Furthermore, the correlation between Sertoli cell number and both testicular size and sperm production is well established (Orth *et al.*, 1988). Sertoli cells divide rapidly and extensively during fetal and early postnatal life, after which their mitotic activity is attenuated. At the beginning of puberty, these cells stop proliferating altogether and thereafter their number is considered to be fixed (Steinberger & Steinberger, 1971; Cotes *et al.*, 1987). If Sertoli cell death is caused by toxicants or any other apoptosis inducing factor, this loss can not be compensated at adult stage. Consequently, germ cells will undergo apoptosis due to inadequate number of Sertoli cells and eventually testicular size and sperm production will be reduced.

Lycopene, a non-provitamin A carotenoid, is synthesized by microorganisms and plants, especially by tomatoes, and it is one of the most potent antioxidants among the dietary carotenoids mainly due to its many conjugated double bonds. The antioxidant activity of lycopene is mainly dependent on its scavenging properties of O_2_^**•**–^ and HO^**•**^. Besides antioxidant activity, nonoxidative mechanisms have been proposed for the role of lycopene in the prevention of cancer, such as regulation of intercellular gap junction communication, hormonal and immune systems and metabolic pathways of xenobiotics (see Rao & Agarwal, 2000; Bhuvaneswari & Nagini, 2005).

Lycopene has a protective effect against testicular toxicity (Atessahin *et al.*, 2006a; Turk *et al.*, 2007), spermiotoxicity (Atessahin *et al.*, 2006a, b), cardiotoxicity (Yilmaz *et al.*, 2005), hepatotoxicity and nephrotoxicity (Yilmaz *et al.*, 2005). Very recently we found that lycopene protected Leydig cellular StAR protein and steroidogenic enzyme expression and confirmed its activity against PCBs (Elumalai *et al.*, 2009). In the present study, lycopene was used to scavenge PCBs-induced ROS and to prevent ROS mediated apoptosis in Sertoli cells. Its effect was assessed by analyzing the H_2_O_2_ level and apoptotic parameters (including apoptotic marker caspase-3 activity).

## Materials and methods

### Chemicals

Aroclor 1254 (PCB mixture) was purchased from Chem Service (Pennsylvania, USA). Lycopene was obtained as a gift from Phytoremedies Biolabs Pvt. Ltd. (Maharastra, India). Dulbecco's Modified Eagle's Medium, Nutrient Mixture, F-12 Ham (DMEM/F-12 – 1:1 mixture), collagenase (type IV), agarose, bovine serum albumin (BSA), deoxyribonuclease (DNase), hyaluronidase, trypsin, trypsin inhibitor, acrylamide, bis-acrylamide, ammonium persulfate, N,N,N’,N’-tetramethylethylene diamine (TEMED) solution were purchased from Sigma-Aldrich Pvt. Ltd. (Missouri, USA). Superscript III reverse transcriptase was purchased from Life Technologies – Invitrogen (New York, USA) and Fast PCR Kit was purchased from KAPA Biosystems (Massachusetts, USA). Rabbit polyclonal anti-Bax, rabbit polyclonal anti-cytochrome c antibodies (Abs) and mouse polyclonal anti-Bcl-2 Abs were purchased from Santa Cruz Biotechnology (Texas, USA). Horse radish peroxidase (HRP) conjugated goat anti-rabbit and mouse anti-goat Abs was purchased from Bangalore GENEI (Bengaluru, India). Goat polyclonal anti-β-actin Ab and primers for PCR were purchased from Sigma-Aldrich Pvt. Ltd. Horse radish peroxidase (HRP) conjugated goat anti-rabbit and mouse anti-goat Abs was purchased from Bangalore GENEI. Methanol and all other chemicals were purchased from Sisco Research Laboratories Pvt Ltd. (Maharastra, India).

### Experimental protocol

Animals were maintained as per the National guidelines and protocols, approved by the Institutional Animal Ethical Committee (IAEC No.03/028/07). Healthy adult male albino rats of Wistar strain (*Rattus norvegicus*) from our animal colony weighing 180–200 g (90 days old) were used for the present study. The animals were housed in clean polypropylene cages and maintained in an air-conditioned animal house with alternating 12h light and dark cycles. The animals were fed standard rat pellet diet and clean drinking water was made available *ad libitum*. The rats were divided into four groups and each group consisted of six animals. Group I rats received corn oil (vehicle control) by intraperitoneal injection (i.p.); Group II rats received lycopene (4 mg/kg/bwt^/^day) by gavage; Group III rats received Aroclor 1254 (2 mg/kg/bwt/day) by i.p.; Group IV rats received Aroclor 1254 (2 mg/kg/bwt/day) by i.p. injection and were simultaneously supplemented with lycopene (4 mg/kg/bwt/day) by gavage. The treatment was given once a day for 30days. Doses and duration were selected according to previous publications (Krishnamoorthy *et al.*, 2011). We employed a single dosing regimen of PCB mixture Aroclor 1254 after standardization of different doses. Twenty-four hours after the last treatment, the rats were decapitated, testes were removed for isolation of SCs. Simultaneously, the cauda epididymidis was collected for spermatozoa isolation.

### Isolation, purification and identification of SCs

SCs were isolated by the method of Anway *et al.* (2003) with some modifications. Six testes from three rats were used for the SC preparation. Testes were removed from the experimental animals and decapsulated. The decapsulated testes were placed in a conical tube containing DMEM, washed twice and allowed to settle. The seminiferous tubules were dispersed by collagenase enzyme solution (0.5 mg/mL DMEM) at 34 °C for 15 min and allowed to settle. The supernatant, which contained interstitial cells, was decanted. The tubules were washed thrice and then incubated in a trypsin enzyme solution (0.5 mg/mL DMEM) at 37 °C for 10 min. After two washes, the tubules were washed for the third time in a solution containing trypsin inhibitor (0.3 mg/mL DMEM). The tubules were then allowed to settle and were incubated in a solution containing a mixture of enzymes (0.1% collagenase, 0.2% hyaluronidase, 0.04% DNase I and 0.03% trypsin inhibitor) at 34 °C for 40 min. The preparation was then centrifuged (500 rpm for 4 min) to pellet SCs and the pellet subsequently washed three times with DMEM. To increase the purity of the SCs, the SC containing pellet was subjected to hypo-tonic shock, the cells were centrifuged at 500 rpm for 4 min and the supernatant decanted. The pellet was resuspended, suspension was filtered through 50-micron pore size nylon mesh, and the cells were then washed thrice with DMEM. After pelleting, the SCs were resuspended in DMEM and counted using hemocytometer. The purified SCs were identified by oil red O staining. Isolated SCs were sonicated with homogenizing (0.1 M Tris-HCl buffer, pH 7.4), Trizol reagent and radio immuno precipitation assay (RIPA) buffer for the biochemical, gene and protein expression analysis, respectively.

### Estimation of hydroxyl radical (HO^•^), hydrogen peroxide (H_2_O_2_), lipidperoxide (LPO) and GSH generation

Hydroxyl radical (HO^**•**^) production was quantified by the method of Puntarulo and Cederbaum (1988). HO^**•**^ level of the samples was expressed as µmoles/min/mg protein. H_2_O_2_ generation was assessed by the spectrophotometric method of Pick and Keisari (1981). H_2_O_2_ content of the samples was expressed as µmoles/min/mg protein. LPO was measured by the method of Devasagayam and Tarachand (1987). Malondialdehyde (MDA) content of the samples was expressed as nmoles of MDA formed/mg protein. GSH level was measured by using the method of Moron *et al.* (1965). The level of GSH in Sertoli cells was expressed as mg per mg protein.

### Analysis of Bad, Bid, Bax, BCl-2, cytochrome c, caspase-8 and -3 expressions by semi-quantitative reverse transcriptase-polymerase chain reaction (RT-PCR)

Total RNA from isolated SCs was extracted using Trizol reagent. Concentration of purified RNA was measured by absorbance at 260 nm. RT-PCR was then performed for Bad, Bid, Bax, BCl-2, cytochrome c, caspase-8 and -3 mRNA expressions. Nucleotide primers used in this study are given in Table 1. Reverse transcription was performed in two steps, in first step Oligo dt (10 µM), dNTP (10 µM), RNA Template (1 µg) and sterile water were taken in a PCR vial and incubated at 65 °C for 5 min and kept in ice for 2 min, the contents were centrifuged and collected. Then 4 µL first strand buffer, 1 µL DTT (0.1 M), 0.2 µL superscript IIIRT were taken in a PCR vial and incubated for 25 °C for 5 min, 50 °C for 45 min and then 70 °C for 15 min and finally maintained at 4 °C for 5 min. The cDNA was amplified by PCR with the help of specific primers. PCR was performed with 35 cycles as follows: 95 °C for 5 min (to activate Taq enzyme), 94 °C for 30 sec (denaturation), 56–57 °C for 30 sec (primer annealing) and 72 °C for 1 min (primer extension) and final extension for 10 min at 72 °C, with 4 °C pause. After PCR, 5.0 µL reaction mix was analyzed on 2% agarose gel with ethidiumbromide. The levels of mRNA were measured by densitometric analysis and standardized by comparison to the GAPDH and β-actin control using a digital imaging and analysis system (Quantity One software).

**Table 1:** The list of primers.

Gene	Sequence	Gene Accession No	Amplified Product (bp)	Annealing Temp/Cycles
Bad	**Sense**: CAGGCAGCCAATAACAGTCA **Antisense**: CCATCCCTTCATCTTCCTCA	NM_022698.1	100	55 °C/35
Bid	**Sense**: GTCATCCACAACATTGCCAG **Antisense**: AGACGTCACGGAGCAGAGAT	NM_022684.1	268	55 °C/35
Bax	**Sense**: GACACCTGAGCTGACCTTGG **Antisense**: GAGGAAGTCCAGTGTCCAGC	NM_ 031632	310	60 °C/35
Bcl2	**Sense**: GGGATGCCTTTGTGGAACTA **Antisense**: CTCACTTGTGGCCCAGGTAT	NM_016993.1	138	55 °C/35
Casp8	**Sense**: GCGACAGGTTACAGCTCTCC **Antisense**: GCAGCCTCTGAAATAGCACC	NM_022277.1	180	55 °C/35
Casp3	**Sense**: AGTTGGACCCACCTTGTGAG **Antisense**: AGTCTGCAGCTCCTCCACAT	NM_012922.2	298	55 °C/35
GAPDH	**Sense**: ACCACAGTCCATGCCATCAC **Antisense**: TCCACCACCCTGTTGCTGTA	NM_017008.2	465	55 °C/35
β-actin	**Sense**: GCCATGTACGTAGCCATCCA **Antisense**: GAACCGCTCATTGCCGATAG	NM_031632	374	58 °C/35

**Bad:** Bcl-2-associated death promoter, **Bid:** BH3 interacting domain death agonist, **Bax:** Bcl-2–associated X protein, **Bcl2:** B-cell CLL/lymphoma 2, **Casp3, 9, 8**: Cysteine- aspartic proteases

### Analysis of Bax, BCl-2 and cytochrome c protein expression by immunoblotting

50 µg of total SC protein was mixed with 2X sample buffer and kept in boiling water bath for 5 min. The sample mixture was run on 12% SDS-PAGE gel in 1X running gel buffer at 80 V and electrotransferred to a PVDF membrane (Millipore, USA) at 100 V for 1 h. The membranes were blocked in blocking buffer containing 5% albumin for an hour. Then the blocked membranes were incubated with rabbit polyclonal anti-Bax Abs (1:2500), mouse polyclonal anti-Bcl-2 (1:1000) for 3 to 6 h and mouse polyclonal anti-β-actin (1:5000) Abs for 3 to 6 h. The membranes were washed and were incubated with horse radish peroxidase (HRP)-labelled goat anti-rabbit and goat anti-mouse IgG Abs for the appropriate primary Abs. Following two intermittent washes with 1X T-TBS and TBS alternatively, the bands were developed using ECL kit (Pierce, Rockford, IL) and intensity of each band was determined using an image analyzer (Quantity One Software from Bio Rad). Immunoblot for β-actin was used as an internal control for equal loading in the gel.

### Caspase-3 Activity

Caspase-3 activity was determined by using assay kit as per the manufacturer's protocol (Biovision, USA). Briefly, equal concentration (50 µg) of protein was added to each well and 50 µl of 29 reaction buffer was added to all the wells. Then 5 µl of DEVD-pNA (Asp-Glu-Val-Asp-p-nitroanilide) was added, incubated for 1 h and the color intensity was read at 400 nm. Fold increase in caspase-3 activity was determined by comparing the results with control.

### Analysis of sperm count and motility

Experimental rats were sacrificed, epididymis were rapidly removed and placed in a saline solution. Spermatozoa were collected from cauda epididymis as described by Seligman *et al.* (1991). Sperm count in cauda epididymis was determined by our laboratory procedure (Krishnamoorthy *et al.*, 2007). Briefly, the cauda epididymis was minced with anatomical scissors in 5ml of physiological saline, placed on a rocker for 10 min and incubated at room temperature for 2 min. The supernatant fluid was diluted (1:100) and the total sperm number was determined with a hemocytometer. Ten µl of the diluted sperm suspension was transferred to each counting chamber, allowed to stand for 5 min and counted with the help of microscope. The sperm motility was determined by the method of Ratnasoorya (1984). Briefly, fluid was collected from cauda epididymis, diluted to 2 ml with Tris buffer solution, prewarmed (35 °C) and percentage of motility was evaluated visually by using a light microscope.

### Statistical analysis

The data were subjected to statistical analysis using one-way analysis of variance (ANOVA) and Student-Newman-Keul's (SNK) test to assess the significance of individual variations between the treatment groups using a computer based software (SPSS 17.5), the significance was considered at the level of *p<*0.05.

## Results

### Effect of lycopene on Sertoli cellular HO•, H_2_O_2_, LPO and GSH in PCBs-exposed adult rats

**Figure 1:**
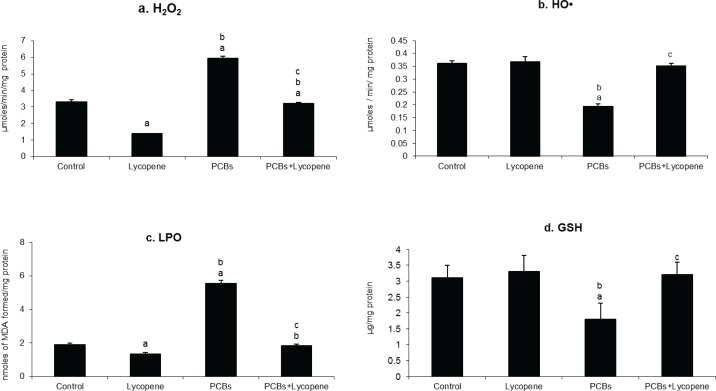
Effect of lycopene on Sertoli cellular H_2_O_2_, HO^•^, LPO and GSH level in PCBs-exposed adult rats. Each bar represents the mean ± SEM of six animals. The statistical significance of the data is *p<*0.05. a-Control Vs others; b-Lycopene Vs PCBs and PCBs+lycopene; c-PCBs vs PCBs+lycopene.

Oxidative stress markers (HO^**•**^, H_2_O_2_ and LPO) levels were estimated and presented in Figure 1. In the present study, HO^**•**^, H_2_O_2_ and MDA levels were significantly increased in Sertoli cells of PCBs-exposed adult rats. Reduced glutathione (GSH) level is one of the best markers to assess the oxidative imbalance within the cell and it is inversely proportional to the level of oxidized glutathione (GS-SG). In this study, GSH level was significantly decreased in SCs of PCBs-exposed adult rats. Oxidative stress was induced by elevated ROS level and reduced antioxidant in SCs. On PCBs exposure H_2_O_2_ and LPO generation were significantly increased in rats. However, simultaneous supplementation of lycopene decreased oxidative stress by preventing ROS production and glutathione oxidation.

### Effect of lycopene on Sertoli cellular caspase-8, Bad and Bid expressions in PCBs-exposed adult rats

**Figure 2:**
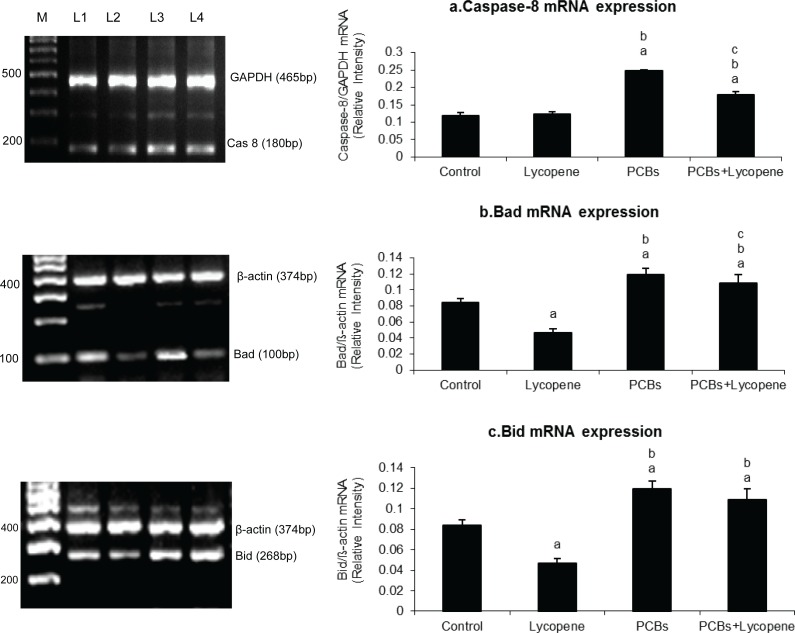
Effect of lycopene on caspase-8, Bad and Bid mRNA expression in Sertoli cells of PCBs-exposed adult rats. M-100bp ladder; L1- control; L2-lycopene; L3-PCBs; L4-PCBs+lycopene. Each bar represents the mean ± SEM of three independent observations. The statistical significance of the data is *p<*0.05. a-Control Vs others; b-Lycopene Vs PCBs and PCBs+lycopene; c-PCBs vs PCBs+lycopene.

Figure 2 shows the level of caspase-8, Bad and Bid mRNA expressions in SCs of PCBs-exposed rats. Caspase-8, Bad and Bid expressions were significantly increased in PCBs-exposed rats. However, simultaneous supplementation of lycopene restored the same parameters to normal when compared with control rats. Sertoli cellular Bid and Bad expressions were decreased in lycopene alone treated rats when compared with control. Caspase-8 mRNA expressions did not alter in Sertoli cells of lycopene alone treated rats.

### Effect of lycopene on Bax and Bcl-2 mRNA and protein expressions in SCs of PCBs-exposed adult rats

**Figure 3:**
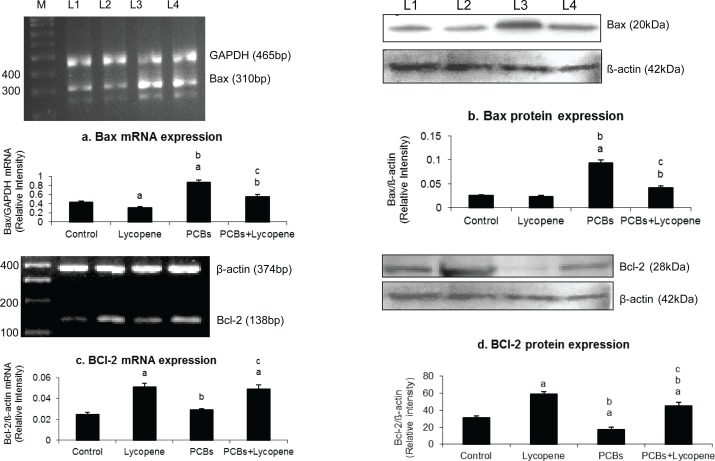
Effect of lycopene on Bax and BCl-2 mRNA and protein expression in Sertoli cells of PCBs-exposed adult rats. M-100bp ladder; L1-control; L2-lycopene; L3-PCBs; L4-PCBs+lycopene. Each bar represents the mean ± SEM of three independent observations. The statistical significance of the data is *p<*0.05. a-Control Vs others; b-Lycopene Vs PCBs and PCBs+lycopene; c-PCBs vs PCBs+lycopene.

Figure 3 shows the effect of lycopene on Bax and Bcl-2 mRNA and protein expressions in SCs of PCBs-exposed adult rats. Bcl-2 and Bax expressions were significantly decreased and increased, respectively. However, simultaneous supplementation of lycopene prevented Bax elevation to avoid apoptosis, which is induced by PCBs. Interestingly, anti-apoptotic protein Bcl2 level was drastically increased in lycopene supplementation.

### Effect of lycopene on cytochrome c release in SCs of PCBs-exposed adult rats

**Figure 4:**
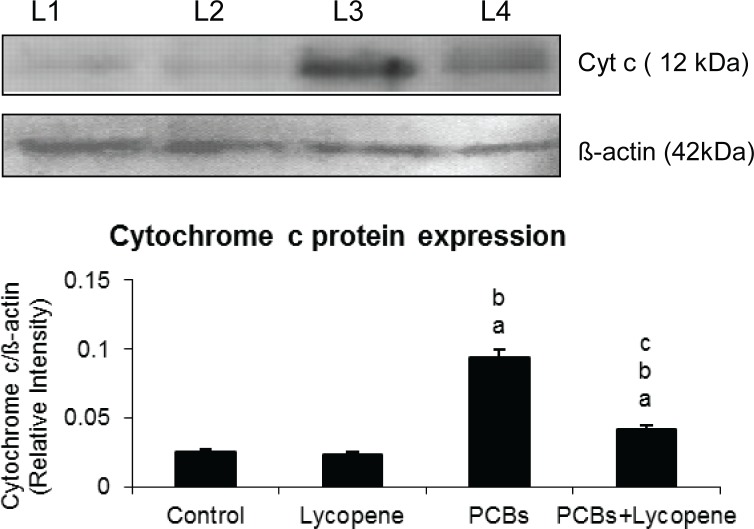
Effect of lycopene on cytochrome c protein expression in Sertoli cells of PCBs-exposed adult rats. L1-control; L2-lycopene; L3-PCBs; L4-PCBs+lycopene. Each bar represents the mean ± SEM of three independent observations. The statistical significance of the data is *p<*0.05. a-Control Vs others; b-Lycopene Vs PCBs and PCBs+lycopene; c-PCBs vs PCBs+lycopene.

Figure 4 demonstrates the cytochrome c level in SCs. On PCBs exposure, cytochrome c expression was significantly increased in Sertoli cells. Simultaneous supplementation of lycopene to PCBs treated rats controlled the cytochrome c expression in SCs. Lycopene alone treatment, did not induce any change when compared with other groups.

### Effect of lycopene on caspase-3 expression and its activity in PCBs-exposed adult rats

**Figure 5:**
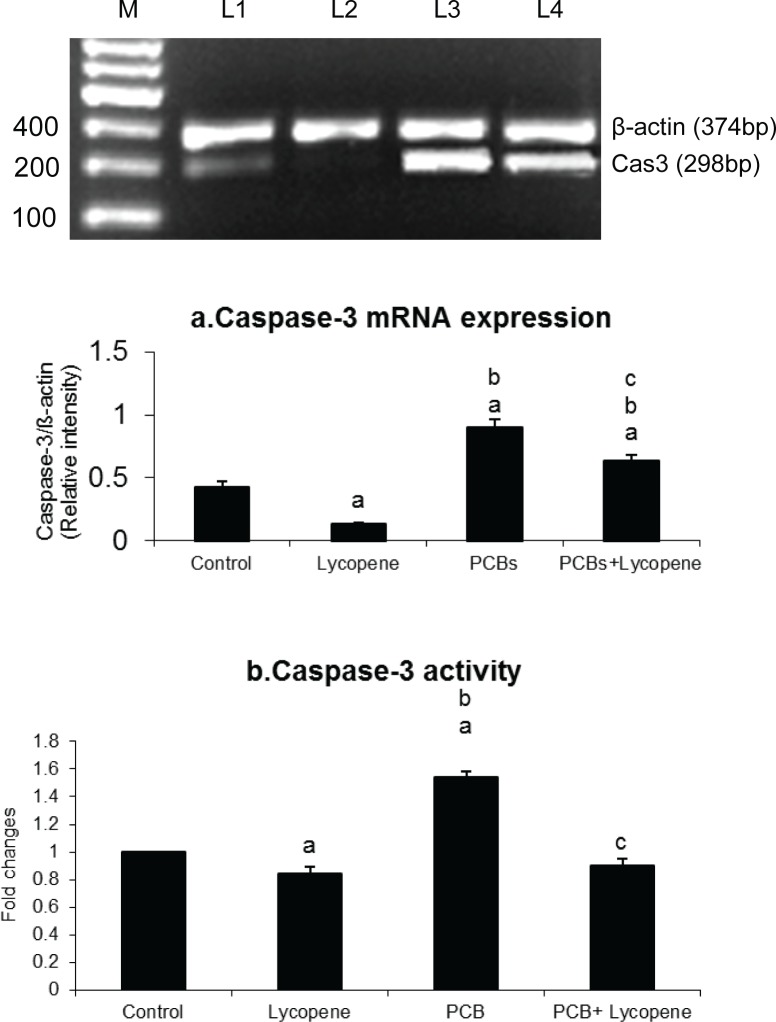
Effect of lycopene on caspase-3 mRNA expression and its activity in Sertoli cells of PCBs-exposed adult rats. M-100bp ladder; L1-control; L2-lycopene; L3-PCBs; L4-PCBs+lycopene. Each bar represents the mean ± SEM of three independent observations. The statistical significance of the data is *p<*0.05. a-Control Vs others; b-Lycopene Vs PCBs and PCBs+lycopene; c-PCBs vs PCBs+lycopene.

Figure 5 depicts the effect of lycopene on Sertoli cellular caspase-3 expression and its activity in PCBs-exposed adult rats. The treatment with PCBs resulted in elevated caspase-3 expression and its activity in SCs of PCBs treated rats. However, simultaneous supplementation of lycopene prevented increased caspase-3 activity, which was close to normal level.

### Effect of lycopene on epididymal sperm count and motility in PCBs-exposed adult rats

**Figure 6:**
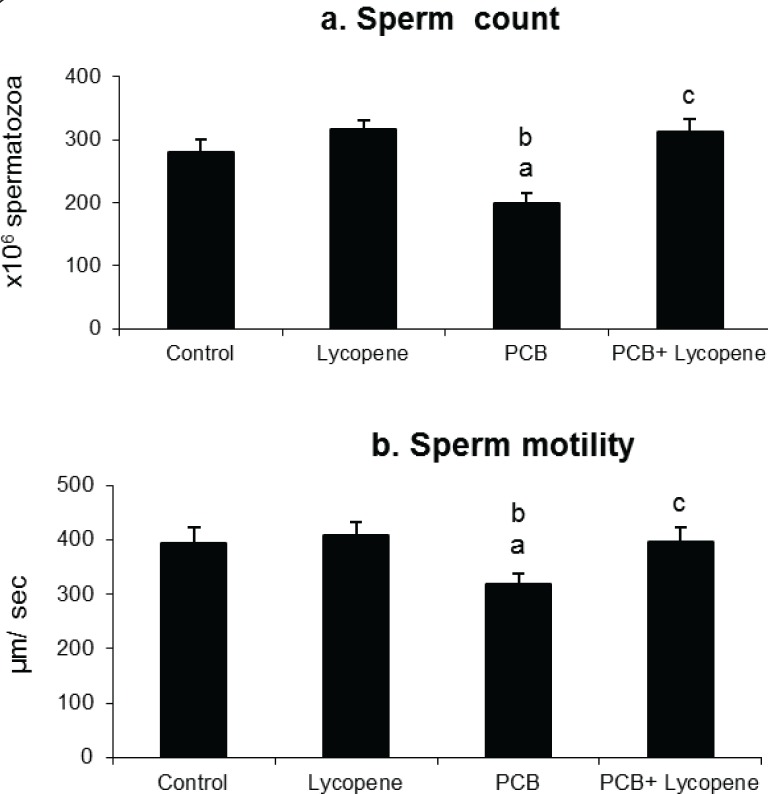
Effect of lycopene on cauda epididymal sperm count and motility in PCBs-exposed adult rats. Each bar represents the mean ± SEM of six animals. The statistical significance of the data is *p<*0.05. a-Control Vs others; b-Lycopene Vs PCBs and PCBs+lycopene; c-PCBs vs PCBs+lycopene.

Figure 6 shows the effect of lycopene on sperm count and motility in SCs of PCBs-exposed adult rats. Sperm count and motility were significantly affected by PCBs-exposure. However, simultaneous supplementation of lycopene protected sperms from PCBs, maintaining normal sperm production and quality. Lycopene alone treatment did not show any significant change in sperm count and motility.

## Discussion

SCs provide the environment and support for GCs during spermatogenesis (Griswold, 1993). SC number is the ultimate determinant of sperm producing capacity and therefore of sperm count. Lycopene has been reported to be effective in prevention of oxidative damage to DNA or to cell membrane (Collins *et al.*, 1998; Porrini *et al.*, 2000; Scolastici *et al.*, 2008a, b).

PCBs are well known to induce apoptotic cell death and to contribute to a variety of pathological conditions. PCBs are known to induce oxidative stress by depletion of glutathione and an increase of reactive oxygen species (ROS) and malondialdehyde (MDA) levels in Leydig cells and SCs (Senthilkumar *et al.*, 2004; Murugesan *et al.*, 2005a, b). Our earlier studies demonstrated that PCBs- induced redox signaling with reference to GSH/GSSG ratio and ROS led to DNA damage and LPO increase in Sertoli cells (Senthilkumar *et al.*, 2004; Krishnamoorthy *et al.*, 2005; 2011) as well as to neurotoxicity (Venkataraman *et al.*, 2006; 2008; 2010). However, studies on apoptotic pathways are lacking. In the present study simultaneous exposure to lycopene blocked the increase in oxidative stress markers and decrease in antioxidants observed in Sertoli cells of PCB exposed rats.

Lycopene is involved in protection of testes against cyclosporine A and adriamycin-associated oxidative damage (Atessahin *et al.*, 2006a, b). However, lycopene reduces toxic effects of PCBs with an antioxidant mechanism that has not been well understood. Several mechanisms have been proposed for the cytoprotective role of lycopene. One mechanism is that lycopene may stabilize lipid membranes and protect LPO by free radical scavenging mechanism, thereby protecting tissues. Alternatively, lycopene may induce hepatocellular metabolic enzymes (Tang *et al.*, 2007) for PCBs, consequently protecting tissues against PCBs-induced toxicity.

Recent literatry sources have suggested that ROS as a signaling molecule can induce caspase family members and play an important role in spermatogenesis and apoptosis (Shi *et al.*, 2009). Oxidative stress seems to be the central element in the regulation of the apoptotic pathways triggered by endocrine disrupting chemicals (EDCs). Previous studies showed that FSH and testosterone were protective for SC survival whereas estradiol induced apoptosis (Yagi *et al.*, 2006). Our recent study showed low levels of FSH and testosterone with increment of ER-β expression in PCBs treated group. We therefore studied the levels of ROS, GSH, expression of apoptotic molecules, activity of apoptotic marker, LDH activity, γ-GT activity, sperm count and sperm motility (Krishnamoorthy *et al.*, 2011).

ROS (H_2_O_2_, HO^**•**^ and LPO) levels were drastically increased in Sertoli cells of PCBs-exposed rats. The increased ROS levels in SCs of PCBs-exposed animals indicated the activation of the cytochrome P450 subfamily through aryl hydrocarbon receptor (AhR). Twaroski *et al.* (2001) have suggested that toxic manifestation induced by PCB may be associated with enhanced production of ROS and thereby induce oxidative stress through the initiation of self-propagating LPO reaction. Beneficially, simultaneous supplementation of lycopene to PCB-treated rats significantly reduced the generation of ROS in SCs when compared with the PCB alone treated group. ROS scavenging activity of lycopene on PCB exposed SCs might be due to superoxide anion and hydroxyl radical attenuation.

Song and Yang (2006) reported that pp-DDE was found to induce DNA damage in SCs which might account for induction of apoptosis. Thus, a direct association between apoptosis induction and increased level of intracellular ROS was investigated in the present study. Caspase-8 expression was found to be significantly increased in PCBs-exposed rats. The reason behind the increased level of caspase-8 is Fas ligand activation due to excess level of ROS. This result is coinciding with an *in vitro* study which demonstrated that endocrine disruptors could induce apoptosis of SCs through a Fas ligand-dependent pathway including nuclear translocation of NF-κB, increased expression of FasL and activation of the caspase-8 (Shi *et al.*, 2009). Oxidative stress induced transcription factors such as NF-κB play a key role in regulation of inflammatory cytokines and adhesion molecule production (De Winther *et al.*, 2005). Binding sites for NF-κB and related transcription factors were identified in promoter regions of inflammatory genes which are up-regulated during PCBs exposure (Shimizu *et al.*, 2007).

One of the primary regulators of mitochondrial mediated pathway to apoptosis is the family of Bcl-2 proteins (Green and Reed, 1998). Bax translocates to the mitochondrial membrane where it forms large multimers that have been suggested to represent the formation of pores in the outer mitochondrial membrane through which cytochrome C and other apoptosis-inducing factors exit into the cytosol (Eldadah & Faden, 2000). In the present investigation, PCBs increased the Bid and Bad expression. In addition, this could result in stimulation of cytochrome c and consequent activation of the caspase cascade (Khan *et al.*, 2000), whereas in the lycopene supplemented group expression of Bad, Bid and cytochrome c was rescued in the PCB-treated rats.

In the presence of cytochrome c and dATP, Apaf-1 (Hengartner 1999; Chinnaiyan 1999) oligomerizes to form an apoptosome complex recruited to the complex by caspase recrand recruits caspase-9, which results in activation of caspase-9 and -3 (Jiang & Wang, 2000). The increased caspase-3 activity in PCB treated groups signifies that PCBs had induced apoptosis which was inhibited during simultaneous lycopene intake. DNA fragmentation is caused by the activity of caspase-3 on a complex of caspase-activated DNase (CAD)/DNA fragmentation factor-40, a nuclease and iCAD/DFF45, its inhibitor (Enari *et al.*, 1998).

In non-apoptotic cells, CAD (caspase activated deoxyribonuclease) is present as an inactive complex with iCAD. During apoptosis, caspase-3 cleaves the inhibitor, allowing the nuclease to cut the chromatin. Blebbing is orchestrated via the cleavage and activation of gelsolin (Kothakota *et al.*, 1997), p21-activated kinase-2 (Lee *et al.*, 1997; Rudel & Bokoch, 1997) and most likely through cleavage of fodrin (Martin *et al.*, 1995) to dissociate the plasma membrane from the cytoskeleton. In total, based on the aforementioned study on the level of ROS and hormonal assay, it was concluded that apoptosis was progressed in PCBs-treated group due to hormonal imbalance of gonadotropin and increased level of ROS. In the present investigation, caspase-3 seems to be the most likely candidate to mediate PCBs-induced apoptosis, as evidenced by the increased protease activity of caspase-3 on PCBs exposure. PCB-induced oxidative stress affected also the testicular architecture and lycopene normalized it (Krishnamoorthy *et al.*, 2012)

Sertoli cells are a major determinant of sperm number in the testis (Orth *et al.*, 1988), while other reproductive cells are involved in sperm motility. PCB-exposure significantly reduced the number and motility of sperms. In the present and earlier studies from our laboratory PCBs-exposure was found to decrease the percentage of viable Sertoli cells in *in vivo* and *in vitro* ( Krishnamoorthy *et al.*, 2005). This could be the main reason for the decreased sperm count in PCBs-exposed animals. Beneficially, simultaneous supplementation of lycopene prevented the spermatogenesis disruption by acting against PCBs.

The present study confirmed that PCBs induceed Sertoli cellular apoptosis by both Fas Ligand and mitochondria mediated pathway, associated with increased oxidative stress. Lycopene prevented the generation of ROS thereby acting against PCBs-induced apoptosis in adult rat SCs. In conclusion, the present study showed that lycopene could provide markable protection against PCBs-induced ROS mediated apoptosis in Sertoli cells.

## References

[1] Anway MD, Folmer J, Wright WW, Zirkin BR (2003). Isolation of Sertoli cells from adult rat testes: an approach to ex vivo studies of Sertoli cell function. Biol Reprod..

[2] Atessahin A, Karahan I, Turk G, Gur S, YIlmaz S, Ceribas AO (2006a). Protective role of lycopene on cisplatin induced changes in sperm characteristics, testicular damage and oxidative stress in rats. Reprod Toxicol.

[3] Atessahin A, Turk G, Karahan I, Yilmaz S, Ceribasi AO, Bulmus O (2006b). Lycopene prevents adriamycin-induced testicular toxicity in rats. Fertil Steril.

[4] Bhuvaneswari V, Nagini S (2005). Lycopene: a review of its potential as an anticancer agent. Curr. Med. Chem. Anticancer Agents.

[5] Carreau S, Foucault P, Drosdowsky MA (1994). Sertoli cells: Functional aspects compared in rats, pigs and man. Ann Endocrinol.

[6] Chinnaiyan AM, O'Rourke K, Tewari M, Dixit VM (1995). FADD, a novel death-domain-containing protein, interacts with the death domain of Fas and initiates apoptosis. Cell.

[7] Cohen LA (2002). A review of animal model studies of tomato carotenoids, lycopene and cancer chemoprevention. Exp Biol Med.

[8] de Winther MP, Kanters E, Kraal G, Hofker MH (2005). Nuclear factor kappaB signaling in atherogenesis. Arterioscler Thromb Vasc Biol.

[9] De Castro BR, Korrick SA, Spengler JD, Soto AM (2006). Estrogenic activity of polychlorinated biphenyls present in human tissue and the environment. Environ Sci Technol.

[10] Devasagayam TP, Tarachand U (1987). Decreased lipid peroxidation in rat kidneys during gestation. Biochem Biophys Res Comm.

[11] Dubois W, Callard GV (1990). Shark testis model: stage-dependent functions and the regulation of spermatogenesis. J Exp Zool.

[12] Eldadah BA, Faden AI (2000). Caspase pathways, neuronal apoptosis and CNS injury. J Neurotrauma.

[13] Elumalai P, Krishnamoorthy G, Selvakumar K, Arunkumar R, Venkataraman P, Arunakaran J (2009). Studies on the protective role of lycopene against polychlorinated biphenyls (Aroclor 1254)-induced changes in StAR protein and cytochrome P450 scc enzyme expression on Leydig cells of adult rats. Reprod Toxicol.

[14] Enari M, Sakahira H, Yokoyama H, Okawa K, Iwamatsu A, Nagata S (1998). A caspase-activated DNase that degrades DNA during apoptosis, and its inhibitor ICAD. Nature.

[15] Green DR, Reed JC (1998). Mitochondria and apoptosis. Science.

[16] Griffith HR, Unswoth J, Blake DR, Lunec J, Rice-Evans (1988). Oxidation of amino acids within serum proteins. Free radicals: Chemistry, Pathology and Medicine Richeliue, London.

[17] Hengartner MO (1999). Programmed cell death in the nematode C. elegans. Recent Prog Horm Res.

[18] Griswold MD (1993). The central role of Sertoli cells in spermatogenesis. Seminars in Cell & Develop Biol.

[19] Hochstein P, Ernster L (1963). ADP-activated lipid peroxidation coupled on TPNH oxidase system of microsomes. Biochem Biophys Res Comm.

[20] Kasai H, Crain PF, Kuchino Y, Nishimura S, Ootsyama A, Tanoaka H (1986). Formation of 8-hydroxy-guanine moiety in cellular DNA by agents producing oxygen radical and evidence for its repair. Carcinogenesis.

[21] Khan SM, Oliver RH, Dauffenbach LM, Yeh J (2000). Depletion of Raf-1 protooncogene by geldanamycin causes apoptosis in human luteinized granulosa cells. Fertil Steril.

[22] Kothakota S, Azuma T, Reinhard C, Klippel A, Tang J, Chu K, McGarry TJ, Kirschner MW, Koths K, Kwiatkowski DJ, Williams LT (1997). Caspase-3-generated fragment of gelsolin: effector of morphological change in apoptosis. Science.

[23] Krishnamoorthy G, Venkataraman P, Arunkumar A, Vignesh RC, Aruldhas MM, Arunakaran J (2007). Ameliorative effect of vitamins (alpha-tocopherol and ascorbic acid) on PCB (Aroclor 1254) induced oxidative stress in rat epididymal sperm. Reprod Toxicol.

[24] Krishnamoorthy G, Selvakumar K, Elumalai P, Venkataraman P, Arunakaran J (2011). Protective role of lycopene on polychlorinated biphenyls (Aroclor 1254)-induced adult rat Sertoli cell dysfunction by increased oxidative stress and endocrine disruption. Biomed Prev Nutr.

[25] Krishnamoorthy G, Murugesan P, Muthuvel R, Gunadharini DN, Vijayababu MR, Arunkumar A, Venkataraman P, Aruldhas MM, Arunakaran J (2005). Effect of Aroclor 1254 on Sertoli cellular antioxidant system, androgen binding protein and lactate in adult rat in vitro. Toxicology.

[26] Martin SJ, O'Brien GA, Nishioka WK, McGahon AJ, Mahboubi A, Saido TC, Green DR (1995). Proteolysis of fodrin (non-erythroid spectrin) during apoptosis. J Biol Chem.

[27] McLean MR, Bauer U, Amaro AR, Robertson LW (1996). Identification of catechol and hydroquinone metabolites of 4-monochlorobiphenyl. Chem Res Toxicol.

[28] Murugesan P, Balaganesh M, Balasubramanian K, Arunakaran J (2007a). Effects of polychlorinated biphenyl (Aroclor 1254) on steroidogenesis and antioxidant system in cultured adult rat Leydig cells. J Endocrinol.

[29] Murugesan P, Senthilkumar J, Balasubramanian K, Aruldhas MM, Arunakaran J (2005a). Impact of polychlorinated biphenyl Aroclor 1254 on testicular antioxidant system in adult rats. Hum Exp Toxicol.

[30] Murugesan P, Muthusamy T, Balasubramanian K, Arunakaran J (2005b). Studies on the protective role of vitamin C and E against polychlorinated biphenyl (Aroclor 1254)-induced oxidative damage in Leydig cells. Free Rad Res.

[31] Nehar D, Mauduit C, Boussouar F, Benahmed M (1998). Interleukin 1a Stimulates Lactate Dehydrogenase A Expression and Lactate production in cultured porcine Sertoli cells. Biol Reprod.

[32] Orrenius S, McCabe MJ, Nicotera P (1992). Ca(2+)-dependent mechanisms of cytotoxicity and programmed cell death. Toxicol Lett.

[33] Orth JM, Gunsalus GL, Lamperti AA (1988). Evidence from Sertoli cell-depleted rats indicates that spermatid number in adults depends on numbers of Sertoli cells produced during perinatal development. Endocrinology.

[34] Pick E, Keisari Y (1981). Superoxide anion and hydrogen peroxide production by chemically elicited peritoneal macrophages-induction by multiple nonphagocytic stimuli. Cell Immunol.

[35] Porrini M, Riso P (2000). Lymphocyte lycopene concentration and DNA protection from oxidative damage is increased in women after a short period of tomato consumption. J Nutr.

[36] Puntarulo S, Cederbaum AI (1988). Effect of oxygen concentration on microsomal oxidation of ethanol and generation of oxygen radicals. Biochem J.

[37] Rao AV, Agarwal S (2000). Role of antioxidant lycopene in cancer and heart disease. J Am Coll Nutr.

[38] Ratnasoorya WD (1984). Effect of atropine on fertility of female rat and sperm motility. Indian J Exp Biol.

[39] Rudel T, Bokoch GM (1997). Membrane and morphological changes in apoptotic cells regulated by caspase-mediated activation of PAK2. Science.

[40] Schlezinger JJ, Keller J, Verbrugge LA, Stegeman JJ (2000). 3,3’,4,4’-Tetrachlorobiphenyl oxidation in fish, bird and reptile species: relationship to cytochrome P450 1A inactivation and reactive oxygen production. Comp. Biochem. Physiol C Toxicol Pharmacol.

[41] Seligman J, Shalgi K, Oschry Y, Kosower NS (1991). Sperm analysis by flow cytometry using the fluorescent thiol labeling agent monobromobimane. Mol Reprod Dev.

[42] Senthilkumar J, Banudevi S, Sharmila M, Murugesan P, Srinivasan N, Balasubramanian K, Aruldhas MM, Arunakaran J (2004). Effects of vitamin C and E on PCB (Aroclor 1254) induced oxidative stress, androgen binding protein and lactate in rat Sertoli cells. Reprod Toxicol.

[43] Shi Y, Song Y, Wang Y, Wang Y, Liang Y, Hu Y, Yu H, Guan X, Cheng J, Yang K (2009). Benzene hexachloride induces apoptosis of rat Sertoli cells through generation of reactive oxygen species and activation of JNKs and FasL. Environ Toxicol.

[44] Shimizu K, Ogawa F, Thiele JJ, Bae S, Sato S (2007). Lipid peroxidation is enhanced in Yusho victims 35 years after accidental poisoning with polychlorinated biphenyls in Nagasaki, Japan. J Appl Toxicol.

[45] Scolastici C, Lopes GA, Barbisan LF, Salvadori DM (2008). Tomato oleoresin inhibits DNA damage but not diethylnitrosamine-induced rat hepatocarcinogenesis. Exp Toxicol Pathol.

[46] Scolastici C, Alves de Lima RO, Barbisan LF, Ferreira AL, Ribeiro DA, Salvadori DM (2008). Antigenotoxicity and antimutagenicity of lycopene in HepG2 cell line evaluated by the comet assay and micronucleus test. Toxicol In vitro.

[47] Song Y, Yang KD (2006). Effect of p, p’-DDE on DNA damage and expression of FasL gene of rat Sertoli cell in vitro. Wei Sheng Yan Jiu.

[48] Steinberger A, Klinefelter GR (1993). Sensitivity of Sertoli and Leydig cells to xenobiotics in in vitro models. Reprod Toxicol.

[49] Steinberger A, Steinberger E (1971). Replication pattern of Sertoli cells in maturing rat testis in vivo and in organ culture. Biol Reprod.

[50] Syed V, Gu W, Hecht N (1997). Sertoli cells in culture and mRNA differential display provide a sensitive early warning assay system to detect changes induced by xenobiotics. J Androl.

[51] Tang L, Guan H, Ding X, Jia-Sheng W (2007). Modulation of aflatoxin toxicity and biomarkers by lycopene in F344 rats. Toxicol Appl Pharmacol.

[52] Tapiero H, Townsend DM, Tew KD (2004). The role of carotenoids in the prevention of human pathologies. Biomed Pharmacother.

[53] Turk G, Atessahin A, Sonmez M, Yuce A, Ceribas AO (2007). Lycopene protects against cyclosporine A-induced testicular toxicity in rats. Theriogenology.

[54] Twaroski TP, O'Brien ML, Larmonier N, Glauert HP, Rorbertson LW (2001). Polychlorinated biphenyls-induced effects of metabolic enzymes, AP-1 binding, vitamin E, and oxidative stress in the rat liver. Toxicol Appl Pharmacol.

[55] Venkataraman P, Selvakumar K, Krishnamoorthy G, Muthusami S, Rameshkumar R, Prakash S, Arunakaran J (2010). Effect of melatonin on PCB (Aroclor 1254) induced neuronal damage and changes in Cu/Zn superoxide dismutase and glutathione peroxidase-4 mRNA expression in cerebral cortex, cerebellum and hippocampus of adult rats. Neurosci Res.

[56] Venkataraman P, Krishnamoorthy G, Vengatesh G, Srinivasan N, Aruldhas MM, Arunakaran J (2008). Protective role of melatonin on PCB (Aroclor 1,254) induced oxidative stress and changes in acetylcholine esterase and membrane bound ATPases in cerebellum, cerebral cortex and hippocampus of adult rat brain. Int J Dev Neurosci.

[57] Wang X (2001). The expanding role of mitochondria in apoptosis. Genes Dev.

[58] Yagi M, Suzuki K, Suzuki H (2006). Apoptotic Sertoli cell death in hypogonadic (hgn/hgn) rat testes during early postnatal development. Asian J Androl.

[59] Yilmaz S, Atessahin A, Sahna E, Karahan I, Ozer S (2006). Protective effect of lycopene on adriamycin-induced cardiotoxicity and nephrotoxicity. Toxicology.

